# Long-Distance FBG Sensor Networks Multiplexed in Asymmetric Tree Topology

**DOI:** 10.3390/s25134158

**Published:** 2025-07-03

**Authors:** Keiji Kuroda

**Affiliations:** Department of Physics, School of Science, Kitasato University, Sagamihara 252-0373, Kanagawa, Japan; kkuroda@kitasato-u.ac.jp

**Keywords:** fiber Bragg grating, tree topology, DFB laser array, time-division multiplexing, wavelength-division multiplexing

## Abstract

This article reports on the interrogation of fiber Bragg grating (FBG)-based sensors that are multiplexed in an asymmetric tree topology. At each stage in the topology, FBGs are connected at one output port of a 50:50 coupler with fibers of different lengths. This asymmetric structure allows the simultaneous interrogation of long-distance and parallel sensor networks to be realized. Time- and wavelength-division multiplexing techniques are used to multiplex the FBGs. Using the heterodyne detection technique, high-sensitivity detection of reflection signals that have been weakened by losses induced by a round-trip transmission through the couplers and long-distance propagation is performed. Quasi-distributed FBGs are interrogated simultaneously, over distances ranging from 15 m to 80 km.

## 1. Introduction

Optical fiber sensors based on fiber Bragg gratings (FBGs) have been studied extensively and are used in various application fields, including structural health monitoring [1], civil engineering [2], and sensing of the natural environment [3]. For these purposes, FBGs offer inherent advantages, including their small size, low losses, light weight, immunity to electromagnetic interference, and design flexibility. The characteristics of fiber sensor networks based on FBGs, e.g., their resolution, sensitivity, multiplexing capability, measurable range, and robustness, must be evaluated for each specific purpose. To enable construction of effective networks, the most fundamental requirement is the multiplexing capability. The number of sensor heads that can be interrogated has been increased using techniques including time-division multiplexing (TDM) [4,5], wavelength-division multiplexing (WDM) [6,7], and a combination of the two methods [8,9].

Long-distance sensing networks are required to realize large-scale health monitoring. Many systems and techniques have been proposed to increase the available sensing distance [10]. Raman amplifiers and erbium-doped amplifiers have been applied in sensor networks [11,12,13]. In these networks, the transmission fibers acted as amplifiers to transmit reflected signals to detection ports with sufficiently high signal-to-noise ratios (SNRs). Multi-wavelength fiber laser schemes that use FBGs as reflectors in cavities have also been studied, to extend the measurable range [14,15]. Using these concepts, the sensing distance has been extended up to 100 km or more. However, the sensing points in these long-distance networks were either singular or limited to operation within narrow ranges. To enable coverage of a large sensing area for long-distance sensing, the distance between FBGs is also an important parameter. In Ref. [16], a Raman amplifier-based interrogation of three FBGs located at 25 km intervals in a single line was reported. In Refs. [17,18], single-bus and double-bus topologies were proposed to interrogate eight FBGs connected in parallel at intervals of 5 km. The double-bus topology was proposed to equalize the reflection intensities from different branches. In addition, the WDM scheme has been used with high-reflectivity FBGs to multiplex sensor heads. This means that the number of sensor heads in the network is determined by the bandwidths of the light sources.

To add robustness to these sensor networks, various network topologies other than the single-line topology have been proposed [19]. One of these topologies is the tree topology, in which the sensor heads are incorporated in independent parallel fiber lines that are connected using optical couplers. Even if a problem occurs at a certain point in one fiber line, the sensor heads in the other fiber lines will still survive. When the number of independent sensor lines increases, the robustness of the system also increases. In contrast, the increase in the number of stages in the topology increases the optical losses. Because of this difficulty, the tree topology has mainly been applied to short-distance sensor networks, to date [20,21]. In a previous paper [22], we proposed a heterodyne detection-based technique that allows high-sensitivity detection of weak reflected signals. In this heterodyne detection (HD) scheme, reflections are observed as amplitudes. Therefore, the reductions in the signal intensity induced both at couplers and during long-distance propagation are reduced when compared with those in the conventional intensity detection (ID) scheme. As a light source, we used a distributed feedback laser (DFB-LD) array, which can emit multi-wavelength pulse trains. This use of the DFB laser array is advantageous for multiplexing of the sensor heads, based on TDM/WDM techniques. Application of the HD technique is suitable for interrogation of networks that suffer large optical losses, including single-line long-distance networks [23] and tree-topology networks [24]. A single-line network is the simplest structure without optical couplers, in which FBG sensor heads are connected in transmission fibers. When trouble occurs at certain position in the fiber line, FBGs cannot be interrogated after the position. The network is relatively fragile and not suitable for constructing a robust network. In tree topology, sensor heads are incorporated in independent parallel fiber lines connected using optical couplers. Thus, the topology can be used to construct a robust sensor system. In symmetric tree topology, all lines are connected at same-stage couplers. Thus, reflection signals have nearly same intensities. Asymmetric tree topology that is studied in this work is a modified structure of the symmetric tree network. The number of sensor lines are smaller than that of the symmetric tree. Meanwhile, long transmission fibers can be inserted before the sensing region. This allows us to construct a long-distance network with ability to cover a large sensing area, from 0 km to 80 km. Because the sensor heads are distributed in parallel lines, robustness of this system is better than that of the single-line network.

This paper reports on the interrogation of FBGs that have been temporally and spectrally multiplexed in an asymmetric tree topology. A five-stage topology containing 50:50 couplers is used to construct long-distance and parallel sensor lines. FBGs are connected to one output port of the coupler at each stage, with delay fibers of different lengths. This asymmetric structure enables realization of simultaneous interrogation of the long-distance and parallel sensor networks. The HD technique is used to increase the SNRs for the weak reflections from the FBGs. A directly modulated DFB-LD array is used as a three-wavelength, frequency-scanning, and interval-controlled pulsed-light source. Quasi-distributed sensing of FBGs placed at distances of 15 m, 20 km, 40 km, and 80 km is performed simultaneously. The rest of the paper is organized follows. Section 2 briefly describes the heterodyne detection technique. Section 3 gives the experimental setup and conditions in detail. Section 4 presents experimental results. Finally, the conclusion is given in Section 5.

## 2. Heterodyne Detection

In HD, a directly-modulated DFB-LD generates pulse trains. These pulse trains are divided into two fields: signal pulses, which are sent to the FBGs, and reference pulses. These pulses have a frequency drift caused by thermal diffusion in the LD. When the optical path length between the signal and reference pulses (which have amplitudes of *E*_sig_ and *E*_ref_, respectively) differs, the interference between the two pulses produces heterodyne beats with an amplitude of 2|*E*_sig_*E*_ref_|. Even in cases where the reflection from the FBG is very weak, the beat amplitude is enhanced by the strong reference amplitude. The beat signal, which is acquired using a balanced detector (BD), is written as

*I* = 2α*R*^1/2^(λ)*E*_sig_*E*_ref_ cos(Δωt − Δϕ), (1)

where *α* is a factor that incorporates *ε*_0_*c*/2, the detection efficiency and gain of the BD, and the total loss in a fiber line. Here, *ε*_0_ and *c* are the permittivity and the speed of light, respectively. Δ*ω* and Δ*ϕ* represent the angular frequency of the beat signal and the phase difference between the two pulse fields, respectively, and *R*(*λ*) is the reflectivity of the FBG. By calculating the area of *I*^2^ over the pulse width,

*I*^2^_sum_ = 2α^2^*R*(λ)*I*_sig_*I*_ref_ Σ[1 + cos(2Δωt − 2Δϕ)], (2)

the FBG’s reflectivity *R*(*λ*) can be defined directly using the area [25]. Application of the HD technique and the use of the BD enhance the signal SNRs obtained by more than three orders of magnitude when compared with intensity detection (ID) [22]. As will be described in the next section, the reduction in the signal intensity induced by the round-trip transmission through the couplers and the long-distance propagation in HD is reduced when compared with that in conventional ID.

## 3. Experimental Section

### 3.1. Interrogator

**Figure 1:**
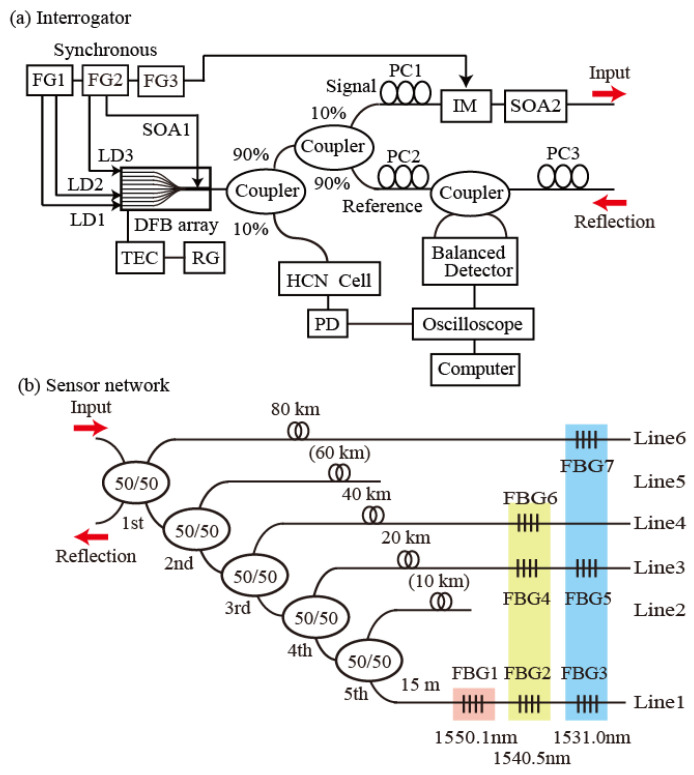
(**a**) Schematic of the experimental setup. LD: laser diode; SOA: semiconductor optical amplifier; DFB array: distributed feedback laser array; FG: function generator; FBG: fiber Bragg grating; PC: polarization controller; PD: photodetector. (**b**) Details of the asymmetric tree-topology sensor network.

Figure 1a shows the interrogator part of the experimental setup. The light source is a DFB laser array that comprises 12 channel LDs, a coupler, and a semiconductor optical amplifier (SOA). The array’s operating range spans the wavelengths from 1530 nm to 1560 nm. The typical output power, the full width at half maximum (FWHM), and the tunable range of each channel are, a few milliwatts, a 5 MHz, and 3 nm, respectively. The optical fields generated by the LDs are amplified by the SOA1. In the experiments, LD1 (operating at 1531.0 nm), LD2 (operating at 1540.5 nm), LD3 (operating at 1550.1 nm), and the SOA are all modulated directly by two synchronized function generators (FG1 and FG2). The DFB array output is divided using a 90:10 coupler. To enable the observation of spectral markers, the 10% proportion of the output is introduced into a H^13^C^14^N fiber-coupled cell. The cell’s transmission is then detected directly, using an amplified photodetector (PD). The other portion of the output is divided again, using a 90:10 coupler to produce signal (10%) and reference (90%) pulses. The signal pulse is shortened using an intensity modulator (IM) controlled via a function generator (FG3), which is synchronized to function generators FG1 and FG2. A polarization controller (PC1) then polarizes the input pulse in a direction parallel to the modulator axis. This shortened signal pulse is then introduced into the sensor network through a circulator, after being amplified by another SOA (SOA2). Because SOA2 is used to amplify the signal pulse, the splitting condition (10% to the signal and 90% to the reference) is chosen in the measurements. The reflections from the FBGs are then combined with the reference pulse, via a 50:50 coupler. To enable effective detection of the beat signals, polarization controller PC2 is used to adjust the polarizations of the signal pulses. The splitting ratio of the 50:50 coupler may not be exactly even, and may have slight wavelength and polarization dependences, which means that the common mode suppression of the reference fields in the BD is imperfect. Therefore, the background levels are not flat when the beat signals are detected. PC3 is used to reduce any undesirable effects by adjusting the polarization state of the reference field. The two coupler outputs are detected using a BD. Finally, the interference signals obtained from the BD and the absorption spectra from the PD are monitored using an oscilloscope; the results are stored on a computer using a LABVIEW (National Instruments, Austin, TX, USA) program, with the sampling rate and the bandwidth being set at 1 GS/s and 300 MHz, respectively. To measure the reflection spectra of the FBGs, the DFB temperature is scanned using a temperature controller that is regulated by a ramp generator, which outputs a ramp voltage with a period of 30 s.

### 3.2. Sensor Network

As shown in Figure 1b, the sensor part has an asymmetric tree topology composed of five 50%/50% couplers. FBG sensing lines and delay fibers are connected at the output ports of the 1st, 3rd, 4th, and 5th couplers. The FBGs have a full width at half maximum (FWHM) of 0.2 ± 0.1 nm, reflectivity of 92%, and sidelobe suppression in excess of 20 dB. The peak wavelengths of the groups (FBG3, FBG5, FBG7), (FBG2, FBG4, FBG6), and FBG1 are 1531.0 ± 0.1 nm, 1540.5 ± 0.1 nm, and 1550.1 ± 0.1 nm, respectively, and correspond to the wavelengths of LD1, LD2, and LD3. Line1 (FBG1, FBG2 and FBG3) is connected at the output port of the 5th coupler. Line3 (FBG4 and FBG5) is connected at the output port of the 4th coupler, with a 20 km delay fiber. The order of the FBGs in each line does not matter. Line4 (FBG6) is connected at the output port of the 3rd coupler, with a 40 km delay fiber. Line6 (FBG7) is connected at the output port of the 4th coupler, with an 80 km delay fiber. Although Line2 and Line5 can be used to construct a larger scale network, we incorporate the seven FBGs in the current experiment. Thus, only four lines, except for the 10 km and 60 km line, are used for the proof of concept. Reflections from the FBGs on the same line are spectrally distinguished. Additionally, reflections from FBGs with the same wavelength in different lines can be distinguished temporally. Because the output from the 1st coupler can be used to extract reflections from the FBGs, a circulator is not used in the current setup.

### 3.3. Dependences of the Reflection Intensities

**Figure 2:**
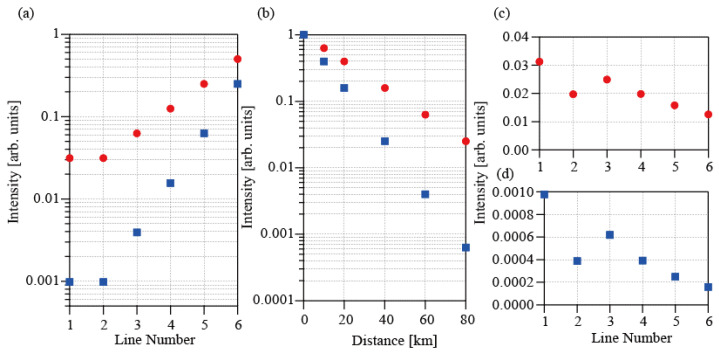
(**a**) Dependences of the normalized signal intensity for ID (red circles) and HD (blue squares) on the number of couplers used. (**b**) Dependences of the normalized signal intensity for ID (red circles) and HD (blue squares) on the propagation distance. (**c**) Total normalized intensity characteristics for HD. (**d**) Total normalized intensity characteristics for ID.

In the long-distance tree-topology network, we must consider losses caused by both the couplers and propagation. Figure 2a shows the dependences of the normalized intensity on the line number for both HD (red circles) and ID (blue squares). The normalized intensity at each coupler is calculated using the intensity (*n* = 0) obtained when the FBG is placed before the 1st coupler. In the ID scheme, the normalized intensity from FBGs connected to an output port of the *n*th coupler is expressed as *y* = (1/2)^2n^ because both the input pulses and the reflected pulses traverse the couplers. In contrast, the normalized intensity in the HD scheme is expressed as *y* = (1/2)^n^ because the reflection is observed as the beat amplitude 2|*E*_sig_*E*_ref_|. The intensity reduction for HD is less than that for ID. Figure 2b shows the dependences of the normalized intensity on the propagation distance for both HD (red circles) and ID (blue squares). The propagation loss for the FBG located at a position of *l* km is expressed as *y* = 10^−2*kl*/10^ in ID. Here, *k* is the propagation loss coefficient of the fibers in dB, and is assumed to be 0.2 dB/km. In contrast, the loss in the HD scheme is expressed as *y* = 10^−*kl*/10^. The intensity reduction for HD is again less than that for ID. Figure 2c,d show the dependences of the total normalized intensity on the line numbers for HD and ID, respectively. The figures show that the dependences on both the coupler number and the distance in Figure 2a,b vary by more than one order, while the change in the total normalized intensity has a value of approximately 3. This result indicates that the reflected signals from FBGs with the same reflectivity located at 15 m and at 80 km can be observed in the same order. In addition, the normalized intensity for the HD case is much higher than that for ID.

### 3.4. Time Charts for the Applied Voltages

**Figure 3:**
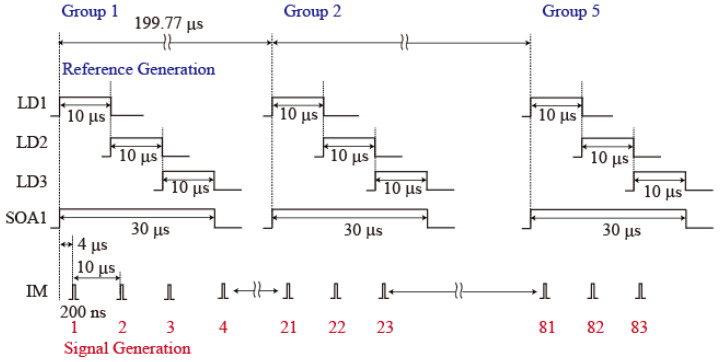
Time charts for the voltage pulses applied to the two LDs, the SOA, and the IM.

**Figure 4:**
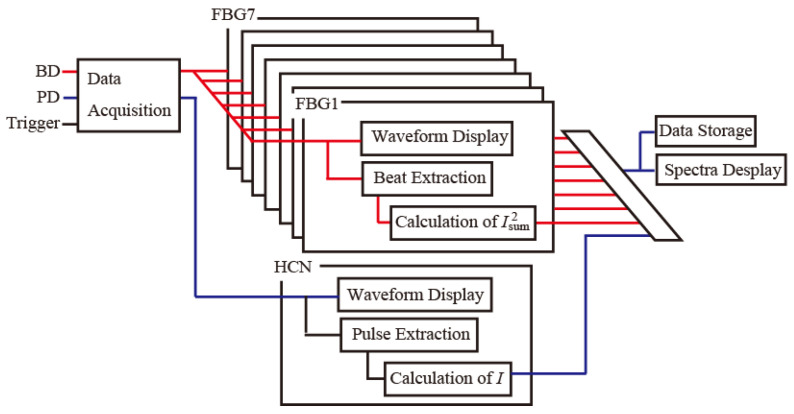
A block diagram of the processing flow. BD: balanced detector, PD: photodetector.

Figure 3 shows time charts for the voltages that were applied to the LDs, the SOA, and the IM. A voltage pulse with a width of 10 µs was applied to LD1 and LD2 from FG1 and to LD3 from FG2. The delay times for LD2 and LD3 were 10 µs and 20 µs, respectively. A voltage pulse with a width of 30 µs was applied to the SOA1 from FG2. These pulses form groups, to generate three wavelength reference pulses. To detect multiple reflections from the FBGs at the different positions simultaneously, five groups of these pulses were generated with an interval of 199.77 µs. 83 pulses with a width of 200 ns at intervals of 10 µs, and, with a delay of 4 µs, were applied to the IM from FG3 to generate the signal pulses. The (*N* − 1) × 20 + 1-th, (*N* − 1) × 20 + 2-th, and (*N* − 1) × 20 + 3-th voltage pulses (where *N* is the number of reference pulse groups) were used to generate three-wavelength signal pulses. As a result, three-wavelength reference pulses with a width of 10 µs and three-wavelength signal pulses with a width of 200 ns were generated. The beat signals of Line1 are observed in the interference between all the signal and reference groups from the same LD output because the path-length difference between the two pulse groups is shorter than the reference pulse width. The beat signals of Line3 are observed in the interference between signal groups 1, 2, 3, and 4 and reference groups 2, 3, 4, and 5. The beat signals of Line4 are observed in the interference between signal groups 1, 2, and 3 and reference groups 3, 4, and 5. The beat signals of Line6 are observed in the interference between signal group 1 and reference group 5. Therefore, all beat signals are observed simultaneously in the interference of reference group 5. The interval between the pulses was set at 1 ms. A block diagram of the processing flow in the LABVIEW program is shown in Figure 4.

## 4. Results

**Figure 5:**
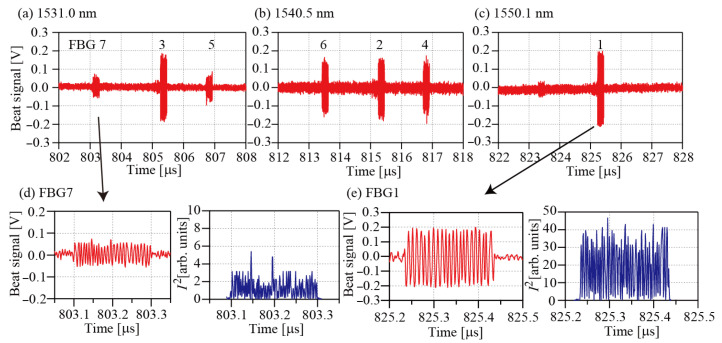
(**a**) Beat signals of FBG7, FBG 3, and FBG 5. (**b**) Beat signals of FBG6, FBG 2, and FBG 4. (**c**) Beat signal of FBG1. (**d**) Left: expanded view of the beat signal of FBG7. Right: *I*^2^ characteristic for the beat signal of FBG7. (**e**) Left: expanded view of the beat signal of FBG1. Right: *I*^2^ characteristic for the beat signal of FBG1.

**Figure 6:**
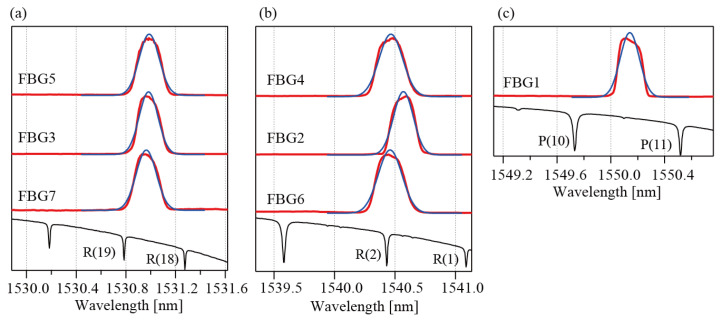
(**a**) Spectra of FBG7, FBG3, and FBG5 with the HCN absorption spectrum. (**b**) Spectra of FBG6, FBG2, and FBG4 with the HCN absorption spectrum. (**c**) Spectrum of FBG1 with the HCN absorption spectrum. The blue lines represent the fitting results.

**Figure 7:**
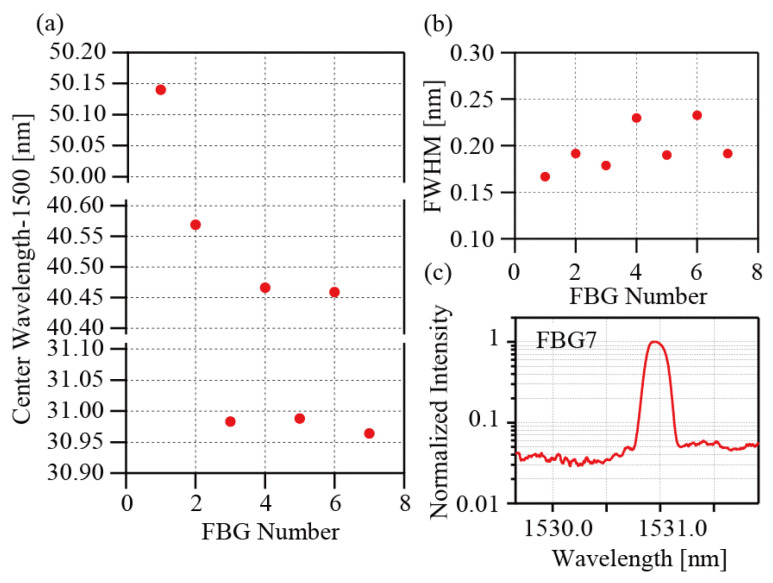
(**a**) Center wavelengths of the FBGs. (**b**) FWHMs of the FBGs. (c) Log-scale plot of the spectrum of FBG7.

Figure 5a–c show the beat signals for the group 5 reference pulses. The beat signals of Line1 appear in the central part of the reference pulses. The beat signals of Line4 and Line6 appear before the signals. The beat signals of Line3 appear after the signals. These random positions occur because the lengths of the delay fibers are not adjusted precisely within the current setup. Interrogation of the FBGs is only possible if all the beat signals appear within the reference pulses. The polarizations possibly change during the propagation. If the reference pulse has a nearly circular polarization, arbitrarily polarized fields can be detected equally. It is expected that reflections from all FBGs in larger-scale systems can be detected by finely adjusting the reference polarization, even when the polarizations of FBG reflections are distributed between linear and circular polarization. The left panels of Figure 5d,e show expanded views of the beat signals of FGB7 and FBG1, respectively. The right panels of Figure 5d,e show the *I*^2^ characteristics for the beat signals. Positive oscillations at twice the beat frequency are observed. The spectra that were calculated from these beat signals are shown in Figure 6a–c. These spectra have been shifted vertically, for clarity. The lower part of each figure shows the absorption spectrum of H^13^C^14^N. Six absorption lines, comprising R(19) at 1530.78615 nm, R(18) at 1531.27537 nm, R(2) at 1540.43120 nm, R(1) at 1541.08703 nm, P(10) at 1549.73051 nm and P(11) at 1550.51546 nm, are used as wavelength markers to determine the peak wavelengths [26]. The abscissas in the figures were calculated using these marker wavelengths. The black lines represent the fitting results that were obtained using a Gaussian function. Generally, the reflection spectra of the low-reflection FBGs can be modeled using Gaussian functions. When the reflectivity increases, however, the reflection spectrum then deviates from the Gaussian function. In this study, FBGs with reflectivity of 92% were used. This high reflectivity accounts for the disagreement between the experimental data and the fitting lines. Therefore, the fitting process was only used to determine the peak wavelengths for the FBGs. In our previous paper, we estimated an accuracy of our method and determined the standard deviation σ to be 3.0 pm [24]. Figure 7a,b show the wavelengths and the FWHMs determined for the FBGs, respectively. All values agree with the specifications (within ±0.1 nm) well. Figure 7c shows a log-scale plot of the spectrum of FBG1, which is the sensor head that was placed at the greatest distance. An SNR of approximately 16 was obtained for this FBG. All SNRs for the other FBGs are better than this value. It is believed that the SNRs are sufficient to determine the peak wavelengths from the spectra in sensing applications.

## 5. Conclusions

This paper has reported on the interrogation of FBGs that have been multiplexed in an asymmetric tree topology. The HD technique is used to increase the SNRs for weak reflections from the FBGs. TDM and WDM techniques are used to increase the number of FBGs in the network. This asymmetric topology allowed us to realize simultaneous interrogation of long-distance and quasi-distributed sensor networks. The topology’s parallel structure added robustness to the system. Although only four lines were used for the proof-of-concept experiment in this work, FBGs in six parallel lines can be interrogated simultaneously by applying direct modulations with an appropriate interval. The DFB laser used in the experiment can emit 11 wavelengths at intervals of 3 nm. Therefore, the construction of a six-line 11-WDM sensor system is not difficult when using our method. Because the DFB laser array was developed for wavelength-division multiplexed communication, successive emission of 11 wavelength pulses with a width of about 5 MHz is possible. Thus, the bandwidth of pulses and delay spread during the propagation is negligible. When 11 FBG fibers are connected in series, transmission loss between 2 fibers may matter. Thus, it is preferable to use a single fiber containing all FBGs with 11 wavelengths for implementation of this system. The resolution of our measurement is achieved by using the molecular markers. The HCN absorption spectra distributes from 1530 nm to 1560 nm. Thus, it is expected that the interrogation of 11 wavelength FBGs can be performed with the same resolution.

## Data Availability

Data can be provided upon request.

## Abbreviations

The following abbreviations are used in this manuscript:

DFB-LD	Distributed feedback laser
FBG	Fiber Bragg grating
TDM	Time-division multiplexing
WDM	Wavelength-division multiplexing
HD	Heterodyne detection
ID	Intensity detection
BD	Balanced detector
SOA	Semiconductor optical amplifier
PD	Photodetector
IM	Intensity modulator
FG	Function generator
PC	Polarization controller
FWHM	full width at half maximum
SNR	signal-to-noise ratio
